# Blue-collar work is a risk factor for developing IgG4-related disease of the biliary tract and pancreas

**DOI:** 10.1016/j.jhepr.2021.100385

**Published:** 2021-10-09

**Authors:** Lowiek M. Hubers, Alex R. Schuurman, Jorie Buijs, Nahid Mostafavi, Marco J. Bruno, Roel C.H. Vermeulen, Anke Huss, Henk R. van Buuren, Ulrich Beuers

**Affiliations:** 1 Department of Gastroenterology & Hepatology, Tytgat Institute for Liver and Intestinal Research, Amsterdam University Medical Centers, location AMC, AGEM, Amsterdam, The Netherlands; 2 Department of Gastroenterology & Hepatology, Erasmus Medical Center, Rotterdam, The Netherlands; 3 Institute for Risk Assessment Sciences, Utrecht University, Utrecht, The Netherlands

**Keywords:** asbestos, autoimmune pancreatitis, IgG4-related cholangitis, occupational, AIP, autoimmune pancreatitis type 1, AUMC, Amsterdam University Medical Centers, EMC, Erasmus Medical Center, IgG4-RD, immunoglobulin G4-related disease, IRC, IgG4-related cholangitis, ISCO, International Standard Classification of Occupations, JEM, job exposure matrix, OR, odds ratio, VDGF, vapors, dusts, gases, and fumes

## Abstract

**Background & Aims:**

Immunoglobulin G4-related disease (IgG4-RD) of the biliary tract and pancreas is a fibroinflammatory disease of unknown origin with striking male predominance. We aimed to investigate whether blue-collar work and occupational contaminant exposure are risk factors for IgG4-RD of the biliary tract and pancreas.

**Method:**

We performed an age-/sex-matched case-control study in the largest academic medical centers of the Netherlands. Occupational history was surveyed by questionnaires. The International Standard Classification of Occupations (ISCO88) was used to classify jobs. Job exposure matrices *ALOHA* and *DOM* were utilized to assess the years individuals were exposed to compounds. The disease control cohort consisted of patients from 6 equally sized groups. Conditional logistic regression was used to assess effects of blue-collar work and exposure to occupational contaminants on developing IgG4-RD of the biliary tract and pancreas.

**Results:**

Overall, 101 patients with IgG4-RD of the biliary tract and pancreas were matched 1:3 to 303 controls. Patients with IgG4-RD had a lower level of education (*p* = 0.001). Individuals who at least once performed blue-collar work (>1 year), had higher odds of developing IgG4-RD than individuals that only performed white-collar work (odds ratio [OR] 3.66; CI 2.18–6.13; *p <*0.0001). Being ever exposed (>1 year) to industrial *ALOHA* (*e.g.* mineral dust; vapors-dust-gases-fumes) and *DOM* compounds (*e.g.* asbestos) resulted in higher odds of IgG4-RD (OR 2.14; 95% CI 1.26–3.16; *p <*0.001 and OR 2.95; 95% CI 1.78-4.90; *p <*0.001, respectively).

**Conclusion:**

Blue-collar work is a risk factor for developing IgG4-RD of the biliary tract and pancreas putatively driven by exposure to selected industrial compounds; this may explain the striking male predominance among patients.

**Lay summary:**

Immunoglobulin G4-related disease (IgG4-RD) causes tumor-like lesions and typically affects middle-aged to elderly men. The background and cause of this disease remain relatively unknown. In this study, we identified blue-collar work as a risk factor for developing IgG4-RD of the biliary tract and pancreas, which may explain the striking male predominance among patients. Furthermore, these results suggest that toxic exposure to occupational contaminants may drive autoimmunity in IgG4-RD of the biliary tract and pancreas.

## Highlights

• Blue-collar work in 68% of people with biliary/pancreatic IgG4-RD vs. 39% of disease controls
• Toxic exposure to selected occupational contaminants may drive autoimmunity in IgG4-RD
• Blue-collar work may explain the striking male predominance in biliary/pancreatic IgG4-RD

## Graphical abstract

**Figure:**
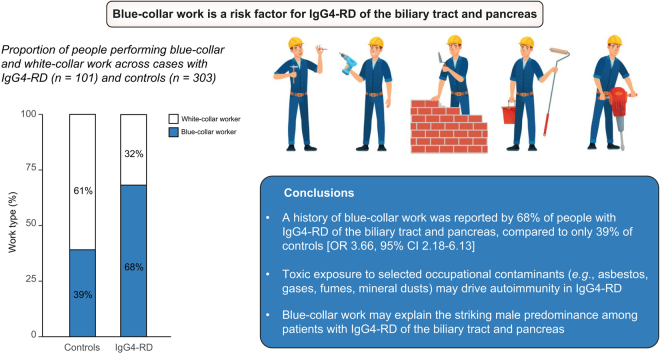

## Introduction

Immunoglobulin G4-related disease (IgG4-RD) is an increasingly recognized multi-organ fibroinflammatory condition.^1^ The various organ manifestations share distinct histological changes, including lymphoplasmacytic infiltrates with large quantities of IgG4-producing plasma cells, a typical storiform pattern of fibrosis and obliterative phlebitis.^2^ The biliary tract and pancreas belong to the organs most frequently affected.^3^^,^^4^ Mass-forming lesions and strictures of IgG4-RD are often difficult to differentiate from pancreatobiliary malignancies and sclerosing cholangiopathies. This diagnostic dilemma leads to major unnecessary surgical and medical interventions for presumed malignancy.^5^ However, if recognized early, lesions may completely disappear upon the start of first-line treatment with predniso(lo)ne.^6^

Insights into the pathogenesis of IgG4-RD remain limited. Auto-reactive B cells are thought to be key players in the disease process, given the fact that selective depletion of CD20+ B cells with rituximab completely reverses signs and symptoms of IgG4-RD.^7^ They may contribute to disease progression through autoantibody formation, antigen presentation and/or cytokine production. Previously, we first described oligoclonal expansions of somatically hypermutated IgG4+ B cells in blood and tissue of patients with IgG4-RD of the bile ducts and pancreas,^8^^,^^9^ and subsequently identified the first IgG4-/IgG1-target auto-antigen Annexin A11.^10^ But it remains unclear what triggers these autoimmune responses in IgG4-RD. Several host factors have been postulated to affect susceptibility to IgG4-RD, including genetic variants,^11^ a history of allergy^12^ or malignancy,^13^ and concomitance of other autoimmune/immune-mediated diseases.^6^ However, these factors do not explain the striking overrepresentation of middle-aged to elderly men among patients with IgG4-RD of the biliary tract or pancreas.^4^^,^^14^ Notably, middle-aged to elderly men account for more than 80% of the patients, which is in remarkable contrast to most other autoimmune diseases.^15^

We previously reported that blue-collar jobs were highly prevalent among an early cohort of our patients with IgG4-RD of the biliary tract and pancreas.^16^ In a pilot study, we then observed that a history of blue-collar work was reported by 88% of patients with IgG4-related cholangitis (IRC) and associated autoimmune pancreatitis type 1 (AIP) in Amsterdam and 61% in Oxford compared to, respectively, 14% and 22% of the disease control patients with primary sclerosing cholangitis.^16^ This observation led us to speculate that exposure to occupational contaminants may have played a role in the development of IgG4-RD of the biliary tract and pancreas. Therefore, we performed an age- and sex-matched occupational case-control study, aiming to challenge the preliminary remarkable findings of our pilot study.^16^ We investigated whether blue-collar work and exposure to occupational contaminants are risk factors for developing IgG4-RD of the biliary tract and pancreas in a cohort of 404 patients.

## Patients and methods

### Design and patient selection

We performed an age- and sex-matched case-control study in the 2 largest academic medical centers and national reference centers for hepatopancreatobiliary diseases in the Netherlands: the Amsterdam University Medical Centers (AUMC, location AMC) and the Erasmus Medical Center (EMC) in Rotterdam. Participants were included between June 2017 and December 2018. Cases comprised patients with IRC and/or AIP meeting the widely used HISORt diagnostic criteria.^4^^,^^14^ A subset of cases were also diagnosed, next to IRC or AIP, with other organ manifestations of the disease (Table S8). Controls were selected from the outpatient clinics of the Departments of Gastroenterology & Hepatology in the same medical centers and consisted of patients from 6 equally sized groups with digestive diseases not known to be provoked by occupational risk factors and not associated with IgG4-RD, including primary sclerosing cholangitis, inflammatory bowel disease, non-alcoholic chronic pancreatitis, malignant and premalignant pancreatobiliary tumors, benign gastrointestinal polyps, and gastrointestinal malignancies. Controls had been referred to the tertiary medical centers for specialized care in the same way as cases. We chose 6 equally sized disease control groups to guarantee a balanced control cohort and to be able to test for potential bias induced by any of the disease control groups (see Table S5). Per hospital, cases and controls were approached similarly. Cases and controls from Amsterdam were invited to participate in the study by the treating physician through an invitation letter. Cases and controls from Rotterdam were approached by telephone on behalf of the treating physician. To increase response rates, non-responders received a reminder phone call after 4 to 6 weeks.

### Questionnaire

We developed a questionnaire with the main objective to collect participants’ complete job history. For every job that lasted more than 1 year, information was asked about job title, dates of employment, average working hours per week, sector of occupation and company name. Furthermore, questions on level of education and lifestyle (consumption of alcohol and tobacco) were posed. The questionnaire also included questions on allergies, malignancies, autoimmune disease and family history. The questionnaire was available in 2 versions, a paper questionnaire and a web-based survey supported by Castor Electronic Data Capture.^17^

### Data collection and handling

Paper surveys were digitalized in Castor EDC, using the unique study ID as identifier and blinded for case-control status.

### Assessment of job history

The International Standard Classification of Occupations (ISCO88)^18^ was used to assess the distribution of white-collar and blue-collar jobs among cases and controls. High-skilled white-collar jobs (ISCO88-major groups 1, 2 and 3) include legislators, senior officials and managers, professionals and technicians and associate professionals; low-skilled white-collar jobs (ISCO88- major groups 4 and 5) include clerks and service workers and shop and market sales workers; high skilled blue-collar jobs (ISCO88-major groups 6 and 7) include skilled agricultural and fishery workers, craft and related trade workers; low skilled blue-collar jobs (ISCO88-major groups 8 and 9) include plant and machine operators and assemblers and elementary occupations. Therefore, all ISCO88-major groups 1-5 were classified as white-collar work, whereas major groups 6-9 corresponded to blue-collar work. All reported jobs were manually coded according to the ISCO88 classification in duplicate by investigators blinded towards case-control status and subsequently recoded to the ISCO68 classification by means of a crosswalk.^19^

### Quantification of exposures

Occupational exposures to a variety of compounds were assigned using job exposure matrices (JEMs). Applied JEMs are semi-quantitative and assign zero, low or high exposure to job codes. For this study, we utilized the JEMs *ALOHA*^20^ and the *DOM*,^21^ both providing exposure scores for a different set of compounds. *ALOHA* assesses exposure to *biological dust, mineral dust, gas fumes, VDGF (vapors, dusts, gases and fumes), all pesticides, herbicides, insecticides, fungicides, aromatic solvents, chlorinated solvents, other solvents,* and *metals*. *DOM* assesses exposure to *asbestos, chromium, diesel motor exhaust, nickel, polycyclic aromatic hydrocarbons, silica, animals, biological dust,* and *endotoxin. ALOHA* uses the ISCO88 codes whilst the *DOM* uses the ISCO68 codes. To calculate the duration of each job employment and thereby the duration of the occupational exposure, both the number of years worked and the hours worked per week were taken into account, yielding a full-time equivalent. Exposures were censored at the time of diagnosis of the patient with IgG4-RD, for both cases and their corresponding controls. In other words, only jobs reported before the date of diagnosis of the cases, and their matched controls, were included in the analyses. To assess exposure to the compounds of interest over time, we calculated the number of years exposed per contaminant for each individual, regardless of the intensity of the exposure.

### Sample size calculation

Prior to the study, a sample size calculation for 2-sample chi-squared test was performed in nQuery based on the pilot study where 74% of all patients with IgG4-RD had a history of blue-collar work.^16^ To detect a minimum odds ratio of 2.5 between cases and controls and thus allowing that a maximum of 54% of controls have a history of blue-collar work, at least 90 cases and 270 controls were needed, when considering an alpha risk of 5%, a power of 90%, and 1:3 matching.

### Matching

Case to control matching was performed using the MatchIt package^20^ in the statistical program R version 3.4.0 in order to have optimally balanced data.^22^ Matching was done using the optimal method, focusing on minimizing the average absolute distance between all pairs based on age (birth year), sex and hospital, in a ratio of 1:3. Standardized mean differences for each covariate were used to judge whether matching improved balance.

### Statistical analyses

Descriptive data are expressed as the mean and standard deviation for continuous variables, and frequency (n) and percentage for categorical variables. *T* test and chi-square tests were used to test for group differences for parametric data, whereas Wilcoxon signed-rank test was used for non-parametric data. Risk was expressed as odds ratio (OR) and 95% CI. Conditional logistic regression was used to assess the effect of blue-collar work and being exposed to occupational contaminants on developing IgG4-RD. In a *post hoc* analysis, comparing cases and controls among blue-collar workers only, we tested whether duration of blue-collar work and duration of exposures to *ALOHA* and *DOM* compounds were related to the risk of IgG4-RD. Furthermore, we analyzed exposures over time to the compounds of interest among cases and controls performing blue-collar work.

### Stratification and sensitivity analysis

The main blue-collar work analysis was stratified by sex and hospital. To correct for potential confounding that may have been introduced by the matching process, we performed a sensitivity analysis by correcting the results for the matching variables (age and sex). Additionally, 1 disease control group was omitted at a time to assess whether it unduly influenced results.

### Ethical

This study was considered exempt from further review (W16_221) by the Institutional Review Board of the Academic Medical Center, University of Amsterdam and was performed according to the principles of the declaration of Helsinki. All authors had access to the study data and reviewed and approved the final manuscript.

### Patient and public involvement

Before the start of the study, the questionnaire was piloted on a small group of patients to verify that questions were clearly formulated and resulted in useful answers. Furthermore, patients were asked to give feedback on the time and effort to complete the questionnaire, which was not seen as a major burden. Patients were not involved in the recruitment and conduct of the study.

## Results

### Study population and matching

**Fig. 1:**
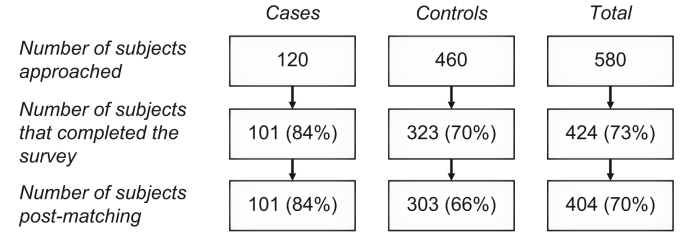
**Flow chart of inclusions**.

A total of 580 patients were approached for this study, of whom 424 patients completed the survey, consisting of 101 cases with IgG4-RD and 323 disease controls (Fig. 1), corresponding to response rates of 84% in cases and 70% in controls. Post-matching, 20 controls were dropped to achieve a 1:3 matching as defined *a priori* and optimal pairs regarding age and sex were formed, resulting in improved standardized mean differences per matching variable (Table 1). Truncating data after time of diagnosis resulted in loss of 3 jobs among control patients from the dataset. However, since these jobs were not their first or only job, no control patient was lost due to truncation. Furthermore, 38 jobs among cases and 151 jobs among matched controls had end dates beyond the diagnosis date (of the corresponding case), and exposure duration was therefore censored at the time of diagnosis of the respective case.

**Table 1:** **Standardized mean differences among covariates pre- and post-matching**.

	Pre-matching			Post-matching		
	Cases (n = 101)	Controls (n = 323)	SMD	Cases (n = 101)	Controls (n = 303)	SMD
Age, mean (SD)	69.2 (10.8)	68.0 (10.7)	0.117	69.2 (10.8)	68.6 (10.3)	0.062
Male sex, n (%)	85 (84.2)	256 (79.3)	0.127	85 (84.2)	253 (83.5)	0.018
Amsterdam UMC, n (%)	71 (70.3)	230 (71.2)	0.020	71 (70.3)	217 (71.6)	0.029

Continuous variables were tested using *t* test and categorical variables were tested using chi-square test.

Amsterdam UMC, Amsterdam University Medical Centers; SMD, standardized mean difference.

Baseline characteristics post-matching are shown in Table 2. Patients with IgG4-RD of the biliary tract and pancreas were on average 69.2 (±10.8) years old. In line with other published reports, the majority of patients with IgG4-RD were male (84.2%). We included 70.3% of the cases in the AUMC. These characteristics were similarly distributed among the disease controls after matching. Overall, cases with IgG4-RD had received a lower level of education than controls. The level of education was low (primary school or secondary/prevocational education) in 47.0% *vs.* 41.1%, intermediate (vocational education) in 32.0% *vs.* 18.9%, and high (higher professional education/university) in 21.0% *vs.* 40.1% of cases and matched controls, respectively. Numbers of smoking pack years and regular alcohol consumption, defined as ≥1 alcoholic drinks per week, were not different between cases and controls. The geographical distribution of cases and controls showed clear overlap (Fig. S1). Both cases and controls lived, as expected, in the western part of the Netherlands.

**Table 2:** **Baseline characteristics post-matching**.

	Cases (n = 101)	Controls (n = 303)	*p* value
Age, mean (SD)	69.2 (10.8)	68.6 (10.3)	0.592
Male, n (%)	85 (84.2)	253 (83.5)	1
Amsterdam UMC, n (%)	71 (70.3)	217 (71.6)	0.899
Erasmus MC Rotterdam, n (%)	30 (29.7)	86 (28.4)	
Education level, n (%)	(n = 100)	(n = 302)	0.001
Low	47 (47)	124 (41.1)	
Intermediate	32 (32)	57 (18.9)	
High	21 (21)	121 (40.1)	
Pack years of smoking, mean (SD)#	16.3 (21.9) (n = 98)	14.1 (21.5) (n = 291)	0.403
Regular alcohol consumption, n (%)#	75 (74.2) (n = 99)	235 (78.1) (n = 301)	0.734

Amsterdam UMC, Amsterdam University Medical Centers; Erasmus MC, Erasmus Medical Center.

# At time of completing the questionnaire. Continuous variables were tested using *t* test and categorical variables were tested using chi-square test.

### Blue-collar work is a risk factor for developing IgG4-related disease of the biliary tract and pancreas

**Fig. 2:**
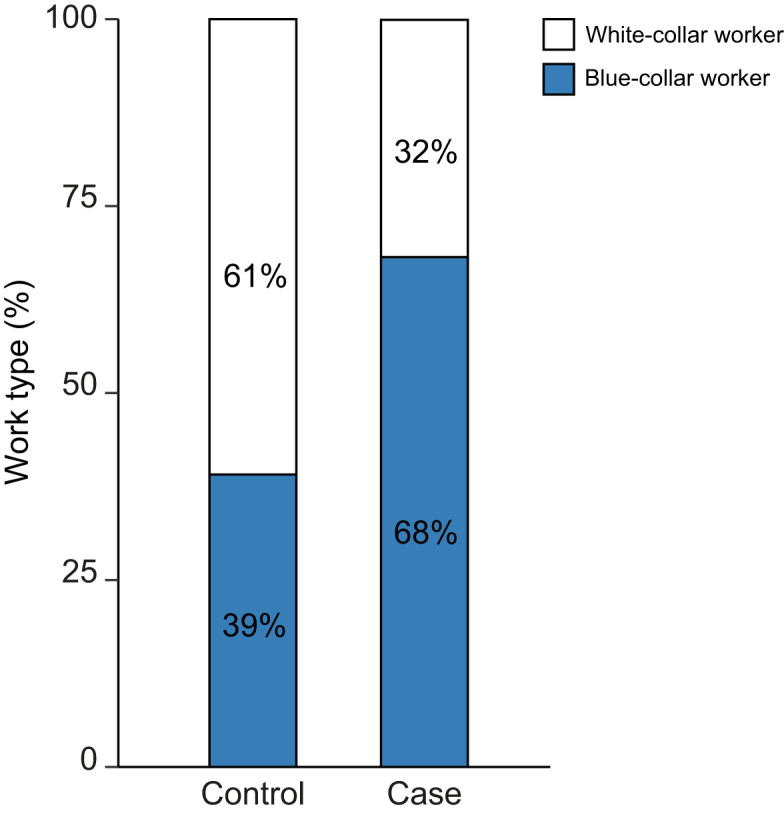
Blue-collar and white-collar work across cases and controls. Proportion of patients performing blue-collar and white-collar work across cases with IgG4-RD of biliary tract and pancreas (n = 101) and disease controls (n = 303) suggesting that blue-collar work (>1 year) is a strong risk factor for IgG4-RD of the biliary tract and pancreas (OR 3.66, 95% CI 2.18–6.13). IgG4-RD, immunoglobulin G4-related disease; OR, odds ratio.

Of cases with IgG4-RD, 68% (n = 69) ever performed a blue-collar job, compared to 39% of controls (n = 117, Fig. 2), corresponding to an OR of 3.66 (95% CI 2.18–6.13). Stratifying by sex (Table S1 and 2) and hospital (Table S3 and 4) yielded similar ORs, which were consistent in direction. To assess whether age, sex, or any specific control group strongly influenced the results of the blue-collar work analysis, we performed a sensitivity analysis for each of these variables (Table S5). In short, neither correction for age, sex, nor the one-by-one removal of the control groups changed the result. *Post hoc* analysis, comparing blue-collar workers only among cases and controls, showed that both groups had comparable characteristics (Table S6). There was a trend towards longer duration of blue-collar work among cases (median 36 years, IQR 15-43 years) compared to controls (median 29 years, IQR 10-40, Wilcoxon signed-rank test *p* = 0.085, Fig. S2), corresponding to a trend towards increased odds of developing IgG4-RD for every 5 years of blue-collar work (OR 1.11; CI 1-1.25; *p* = 0.06).

Taken together, a history of blue-collar work is strongly associated with IgG4-RD of the biliary tract and pancreas, whereas duration of blue-collar work might be related to this risk of developing IgG4-RD.

### Occupational exposure to industrial compounds is a risk factor for developing IgG4-RD of the biliary tract and pancreas

Using the *ALOHA* and *DOM* scores, we investigated whether occupational exposure to industrial compounds differed between cases and controls. In cases, 78% ever had a relevant exposure for at least 1 year to any of the *ALOHA* compounds and 73% to any of the *DOM* compounds, compared to 62% and 48% of the controls, respectively. Conditional logistic regression indicated that individuals who were exposed to any of the *ALOHA* and *DOM* compounds, at least once in their working life, had 2.14- (95% CI 1.26–3.16, *p <*0.001) and 2.95- (95% CI 1.78–4.90, *p <*0.001) fold higher odds of developing IgG4-related disease compared to individuals who were never exposed to industrial compounds in their professional life. Furthermore, cases with blue-collar work (*ALOHA* median = 37 years, IQR = 18-32 years; *DOM* median = 36 years, IQR = 17-45 years) showed a trend towards longer exposure to *ALOHA* compounds and were exposed to *DOM* compounds for significantly longer during their career compared to control blue-collar workers (*ALOHA* median = 32 years, IQR = 14-42 years, Wilcoxon signed-rank test *p* = 0.10; *DOM* median = 30 years, IQR = 9-39 years, Wilcoxon signed-rank test *p* = 0.01, Figs S3 and S4). Using logistic regression, we then investigated whether the duration of exposure to *ALOHA* and *DOM* compounds related to an increased risk of developing IgG4-RD among blue-collar workers. For every 5 years of exposure to any of the *ALOHA* and *DOM* compounds, the odds of developing IgG4-RD increased by 15% (OR 1.15; 95% CI 1.01–1.30, *p* = 0.03) and 14% (OR 1.14; 95% CI 1.02–1.27; *p* = 0.03), respectively. Thus, occupational exposure to industrial compounds might be driving the increased risk of developing IgG4-RD among blue-collar workers, and the duration of exposure is a significant factor herein.

### Occupational exposure to specific compounds influences the odds of developing IgG4-RD

Finally, we investigated whether it was possible to pinpoint specific compounds that could lie beneath this increase in risk of developing IgG4-RD. To this end, we compared exposure to several compounds over time among cases and controls in the blue-collar work group. We found that exposure to several compounds led to an increased OR for developing IgG4-RD (Fig. S3, Table S5), including *ALOHA* compounds (*mineral dusts* and *VDGF),* and the *DOM* compound *asbestos.* In summary, a number of industrial compounds (dusts and fumes), mostly taken up through inhalation, could be associated with the attributable risk of blue-collar work on the development of IgG4-RD of the biliary tract and pancreas.

## Discussion

In this occupational case-control study in 404 patients, we showed that blue-collar work is a significant risk factor for developing IgG4-RD of the biliary tract and pancreas, putatively driven by occupational exposure to specific industrial compounds.

Overall, a history of blue-collar work was reported in 68% of patients with IgG4-RD of the biliary tract and pancreas, while only 39% of matched disease controls ever performed blue-collar work for more than 1 year. In our case-control study, the odds of developing IgG4-RD among blue-collar workers were 3.66-fold higher than among white-collar workers (95% CI 2.18–6.13; *p <*0.001). Among blue-collar workers, the duration of blue-collar work tended to increase the risk of IgG4-RD and the duration of exposure to both *ALOHA* and *DOM* compounds clearly increased the risk of IgG4-RD. We found different exposures over time to *mineral dusts, VDGF* and *asbestos* between patients with IgG4-RD and disease controls even among blue-collar workers, underlining a putative driving role for occupational exposures in the attributable risk of blue-collar work for developing IgG4-RD.

A strength of our study is that we included a large group of patients with IgG4-RD and matched controls in the 2 major tertiary hospitals and hepatopancreatobiliary expert centers in the Netherlands. Cases overall had a lower educational level than controls, which can be explained by the fact that blue-collar workers tend to have a lower level of education compared to white-collar workers. Nevertheless, correcting the main analysis for education did not influence results. When stratified per hospital, the prevalence of blue-collar work was comparable between cases in AUMC (70%) and Rotterdam EMC (63%). However, blue-collar work was more common in the EMC (52%) than in the AUMC (33%) among controls. This difference can be explained by differences in the composition of the population. In general terms, Rotterdam houses the largest continental European harbor with a large industrial zone, attracting a working population of variable skills, whereas Amsterdam as capital of the Netherlands (Den Hague being the seat of the government) is mostly a knowledge, finance and business center drawing highly educated professionals. National statistics performed by the Central Bureau of Statistics (CBS) of the Netherlands in 2011 support this distinction, showing that among the adult population in Rotterdam 42% had a low education level, 35% an intermediate level and 23% a high level, compared to 29%, 31% and 40%, respectively, in Amsterdam.^23^

We aimed to reduce several biases occupational epidemiology studies are prone to.^24^ For practical reasons, we performed a hospital-based, and not a population-based, case-control study. However, controls were representative of the source population that produced the cases, as they were referred to the 2 hospitals for tertiary care in a similar way as cases. This is also supported by the comparable geographical distribution of cases and controls across the country (Fig. S1). Furthermore, 6 well-defined disease control groups – with no known relation to IgG4-RD or occupation – were chosen to balance possible unobserved confounders among controls. Leaving out 1 disease group at a time did not influence results, confirming the lack of relevant confounders in the control groups. Despite consistent approaching-techniques, we achieved a response rate of 84% among cases and 70% among controls. This difference might be explained by the notion that patients could be more likely to participate in research as disease severity increases. For example, the response rate of patients with premalignant or malignant pancreatobiliary disease was 85% compared to only 63% among patients with benign gastrointestinal polyps. Finally, although JEMs are currently among the best validated tools to quantify occupational exposures, it remains challenging to directly link specific exposures to certain disease states. As is often the case in occupational epidemiology, more molecular and cell biological mechanistic research is needed to challenge or confirm these associations.

In this study, we also performed a *post hoc* analysis among cases and controls with blue-collar work only. Although matching was broken inevitably, both groups were comparable in terms of age and sex. We found that exposure to *mineral dusts, VDGF* and *asbestos* led to increased ORs of developing IgG4-RD. Notably, *asbestos* was found to be a risk factor for idiopathic retroperitoneal fibrosis, which is nowadays known as a manifestation of IgG4-RD, in 2 large case-control studies.^25^^,^^26^ Furthermore, various case-reports have already reported on IgG4-RD in asbestos workers.[27], [28], [29], [30] However, *mineral dusts* and *VDGF* have not been related to IgG4-RD yet. How occupational exposure to these industrial compounds contributes to developing IgG4-RD remains subject for further research. The delay between occupational exposure and the development of disease/symptoms in IgG4-RD suggests that effects outlast exposure. Both biochemical and immunological pathogenetic processes are most probably involved.^24^ For instance, toxic substances may cause direct damage to tissues, resulting in release of autoantigens that can induce B cell responses. Tissues may also be targeted by auto-reactive B and T cells upon alterations of the structure of self-proteins by the toxic substance, or by a process of molecular mimicry. In this model, toxic substances may resemble self-protein subunits causing cross-reactivity. Other possibilities are that toxic substances directly affect immune cells through genetic and epigenetic changes or through generation of free radicals. Nevertheless, the result is a chronic inflammatory state that involves many different immune cells. In this situation we believe that IgG4 antibodies – known for their anti-inflammatory properties^31^^,^^32^ – are formed by highly expanded and affinity-matured B cell clones^8^^,^^9^^,^^33^ to dampen an uncontrolled auto-inflammatory response. In line with this hypothesis, we have recently shown that IgG4 blocks binding of IgG1 to the IgG4/IgG1 auto-antigen Annexin A11 which we recently identified in patients with IgG4-RD.^8^^,^^9^ Moreover, patient IgG4 mitigated the pathogenic effects of IgG1 when injected simultaneously in a humanized mouse model, suggesting an anti-inflammatory role for IgG4.^34^

Collectively, we report that blue-collar work is a risk factor for developing IgG4-RD of the biliary tract and pancreas, possibly driven by long-term occupational exposure to a variety of industrial compounds. These findings challenge the current paradigm of IgG4-RD, possibly explaining the striking male predominance among patients with IgG4-RD of the biliary tract and pancreas. This study opens up new avenues for further research on the pathophysiology of this disease. Future collaborative research efforts should try to further unravel the possible relationship between long-term toxic exposure and autoimmunity on the molecular level in IgG4-RD.

## Financial support

The study was supported by grants from the ‘10.13039/501100008985German Morbus Crohn & Ulcerative colitis patient organization (DCCV)’, section PSC (to UB), and the Netherlands Gastroenterology & Hepatology Foundation (MLDS) (to UB).

## Authors’ contributions

L. Hubers: Conceptualization, Data curation, Formal Analysis, Investigation, Methodology, Project administration, Visualization, Writing – original draft. A. Schuurman: Conceptualization, Data curation, Formal Analysis, Investigation, Methodology, Project administration, Visualization, Writing – original draft. J. Buijs: Data curation, Investigation, Project administration, Writing – review & editing. N. Mostafavi: Formal Analysis (Statistician), Methodology, Validation, Writing – review & editing. M. Bruno: Resources, Writing – review & editing. R. Vermeulen: Methodology (Risk assessment), Software, Writing – review & editing. A. Huss: Conceptualization, Methodology (Risk assessment), Software, Writing – review & editing. H. van Buuren: Resources, Writing – review & editing. U. Beuers: Conceptualization, Acquisition of the financial support for the project leading to this publication, Methodology, Project administration, Resources, Validation, Supervision, Writing – review & editing.

## Data availability statement

The anonymized data that support the findings of this study are available on reasonable request from the corresponding author.

## Conflict of interest

The authors have declared that no conflict of interest exists related to this work.

Please refer to the accompanying ICMJE disclosure forms for further details.
