# Supplementary material for: Blue-collar work is a risk factor for developing IgG4-related disease of the biliary tract and pancreas
Source: JHEP Rep. 2021 Oct 9;3(6):100385. doi: 10.1016/j.jhepr.2021.100385 (PMC8593662; doi:10.1016/j.jhepr.2021.100385)
Supplement: Multimedia component 1 [file mmc1.pdf]

Blue-collar work is a risk factor for developing IgG4-related disease of the biliary tract and pancreas

Lowiek M. Hubers, Alex R. Schuurman, Jorie Buijs, Nahid Mostafavi, Marco J. Bruno, Roel C.H. Vermeulen, Anke Huss, Henk R. van Buuren, Ulrich Beuers

Table of contents

Fig. S1..........2
Fig. S2..........3
Fig. S3..........4
Fig. S4..........5
Table S1..........6
Table S2..........6
Table S3..........7
Table S4..........7
Table S5..........8
Table S6..........8
Table S7..........9
Table S8..........10

Fig. S1.

Geographical distribution of cases and controls across the Netherlands.

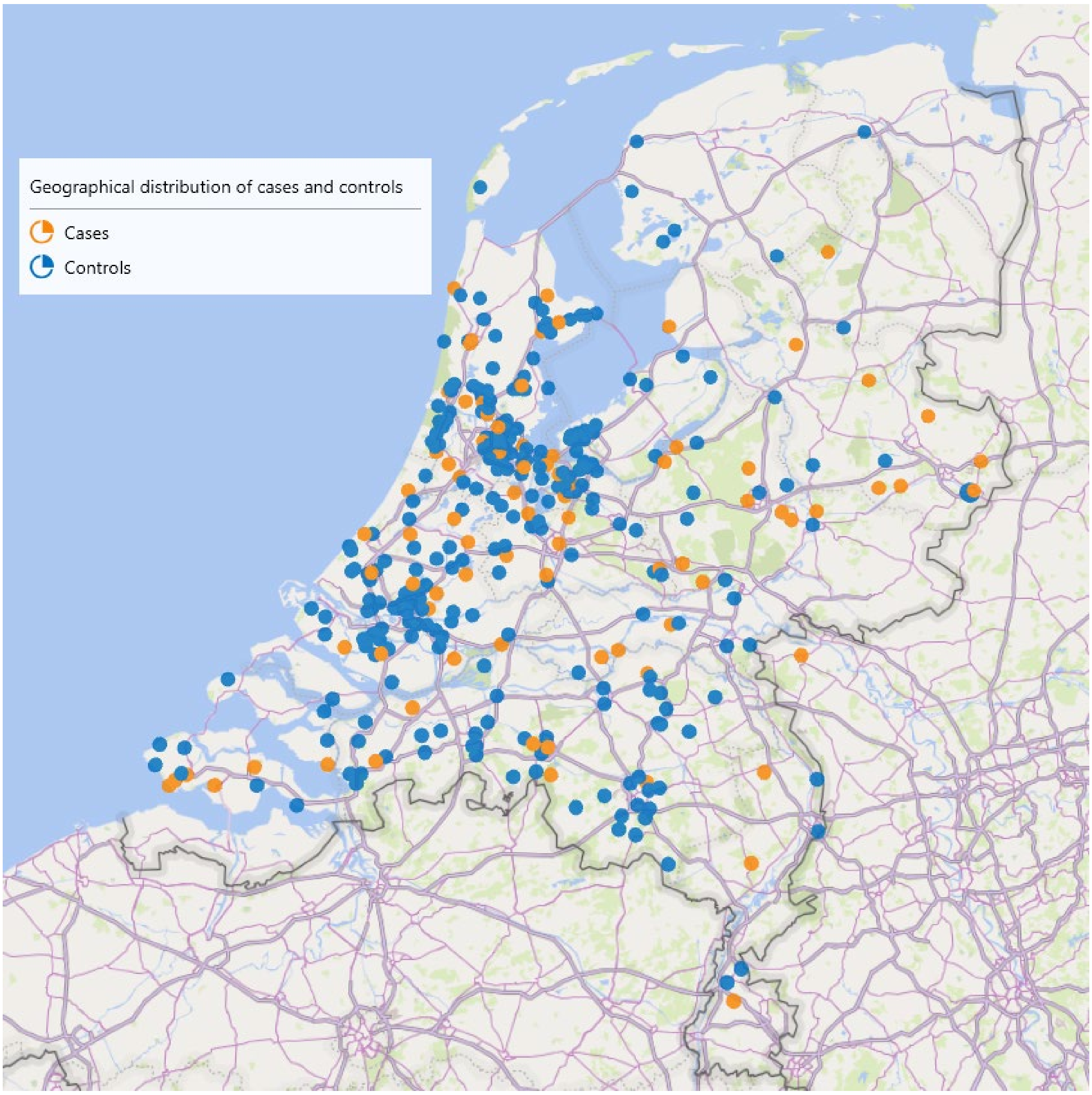

Fig. S2.

*Post hoc analysis among blue-collar workers only, showing the distribution of the number of years cases and controls performed blue-collar work. Significance is determined by the Wilcoxon signed-rank test. * P<0.05 ** P<0.01.*

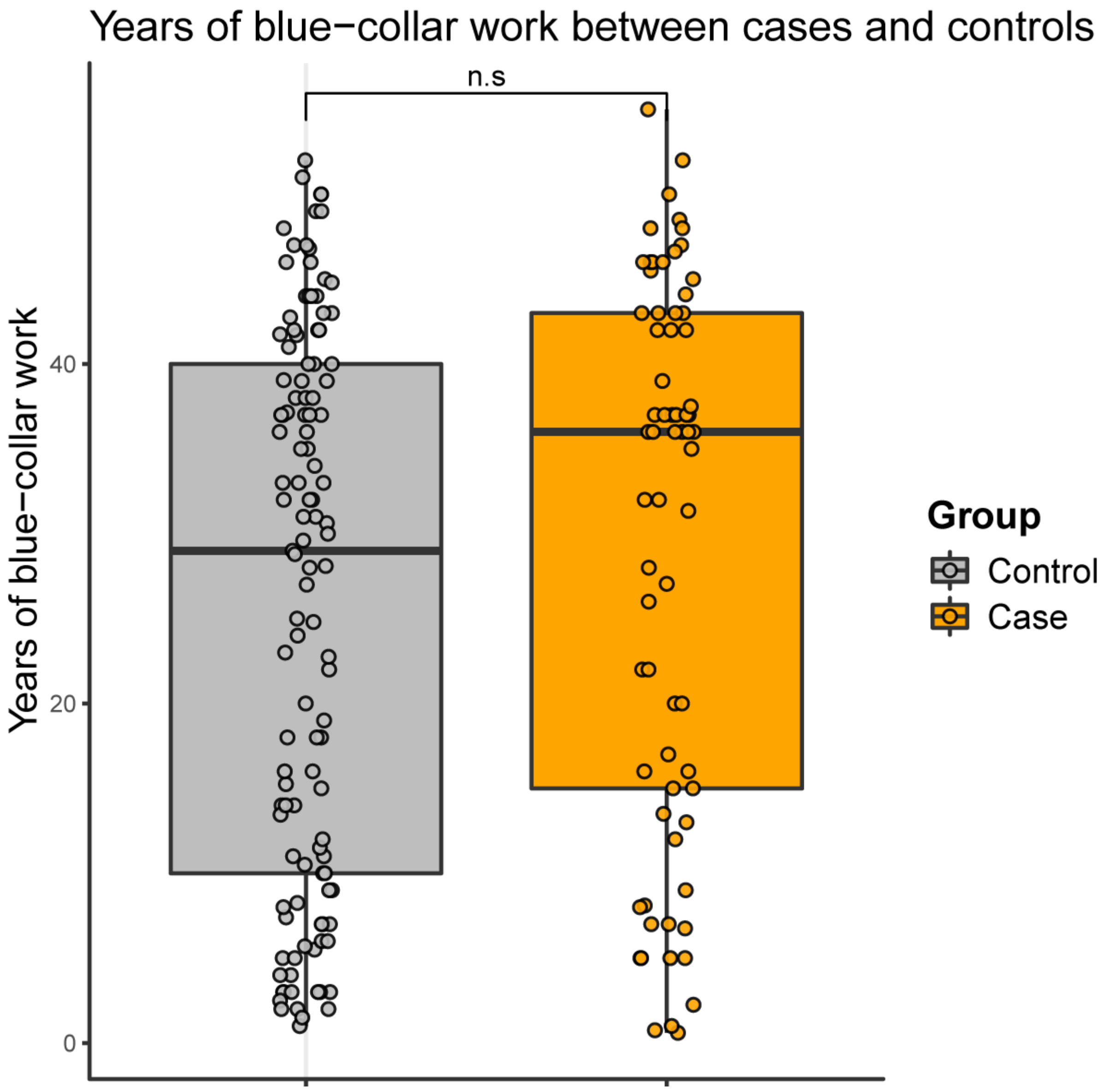

Fig. S3.

*Post hoc analysis among blue-collar workers only, showing the distribution of the number of years cases and controls were exposed to ALOHA contaminants. Significance is determined by the Wilcoxon signed-rank test. * P<0.05 ** P<0.01.*

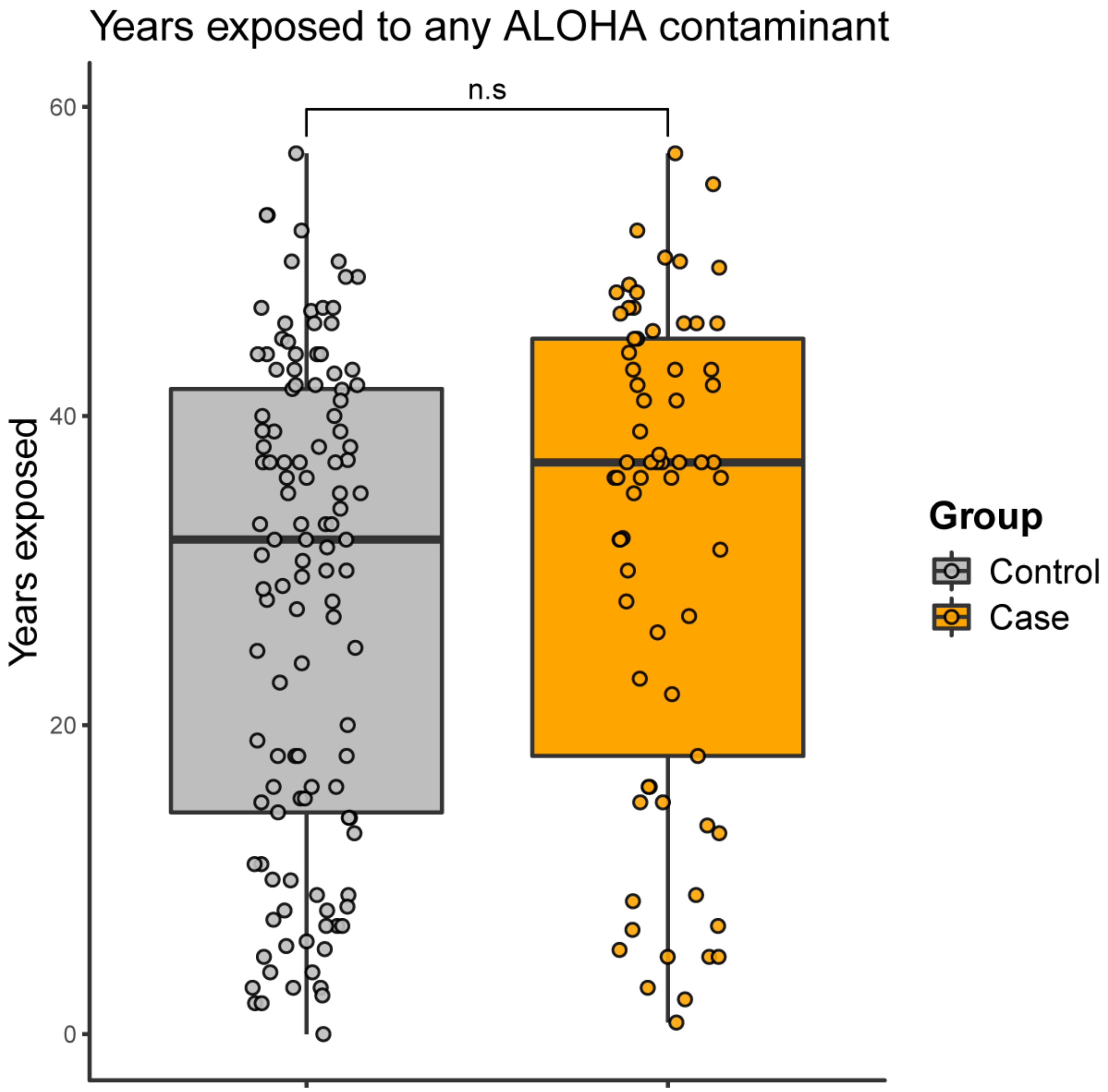

Fig. S4.

*Post hoc analysis among blue-collar workers only, showing the distribution of the number of years cases and controls were exposed to DOM contaminants. Significance is determined by the Wilcoxon signed-rank test. * P<0.05 ** P<0.01.*

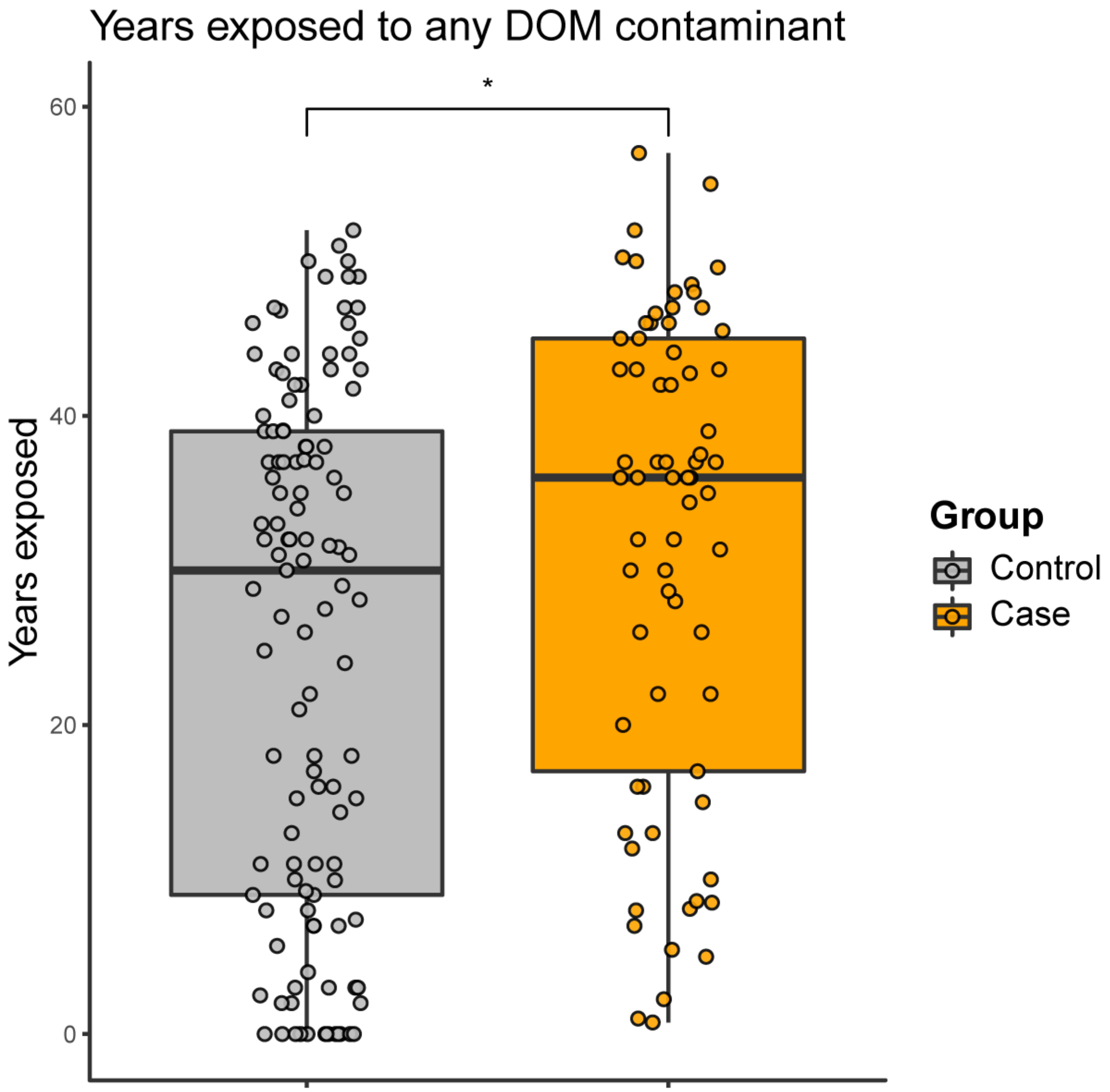

Table S1

Proportion of blue-collar and white-collar work among male and female subjects.

-	Male subjects		Female subjects	
	Blue-collar work	White-collar work	Blue-collar work	White-collar work
Cases (*n (%)*)	65 (76)	20 (24)	4 (25)	12 (75)
Controls (*n (%)*)	110 (43)	143 (57)	7 (14)	43 (86)

Table S2

Odds ratios (OR) and 95%-confidence intervals for effect of having a history of blue-collar work on developing IgG4-RD using conditional logistic regression stratified by gender.

	OR	95% CI	P value
Male subjects	3.69	2.1 – 6.49	<0.0001
Female subjects	2.63	0.42 – 16.42	0.3

Table S3

Proportion of blue-collar and white-collar work among AUMC and EMC subjects.

-	AUMC subjects		EMC subjects	
	Blue-collar work	White-collar work	Blue-collar work	White-collar work
Cases *n (%)*	50 (70)	21 (30)	19 (63)	11 (37)
Controls *n (%)*	72 (33)	145 (67)	45 (52)	41 (48)

AUMC=Amsterdam University Medical Centers, EMC=Erasmus Medical Center, Rotterdam

Table S4

Odds ratios (OR) and 95%-confidence intervals for effect of having a history of blue-collar work on developing IgG4-RD using conditional logistic regression stratified per hospital.

	OR	95% CI	P value
AUMC subjects	6.36	3.04 – 13.28	<0.0001
EMC subjects	2.22	0.53 – 9.27	0.27

AUMC=Amsterdam University Medical centers, EMC=Erasmus Medical Center

Table S5

Odds ratios (OR) and p-values of the blue-collar work analysis after correction for each correction variable.

Correction variable	Corrected OR	P value
Education level	3.04	<0.0001
Hospital	3.67	<0.0001
Gender	3.7	<0.0001
Age	3.91	<0.0001
Pancreatobiliary tumors group omitted	5.63	<0.0001
Colon carcinoma group omitted	3.52	<0.0001
IBD group omitted	2.84	<0.0001
Chronic pancreatitis group omitted	4.59	<0.0001
Gastrointestinal polyps group omitted	3.92	<0.0001
PSC group omitted	2.72	<0.0001

IBD=inflammatory bowel disease, PSC=primary sclerosing cholangitis

Table S6

Characteristics of blue-collar workers

	**Control (n=117)**	**Case (n=69)**	***p***	**SMD**
Male gender (n (%))	110 (94.0)	65 (94.2)	NA	0.008
Age (mean (SD))	70.5 (10.0)	69.6 (10.0)	0.569	0.087
Hospital = EMC (%)	45 (38.5)	19 (27.5)	NA	0.234

EMC=Erasmus Medical Center

Table S7

Post hoc analysis among blue-collar workers only showing odds ratios (OR) and 95%-confidence intervals for effect of being exposed to each contaminant for 5 years on developing IgG4-RD, using conditional logistic regression. A = ALOHA contaminants, B = DOM contaminants.

A. ALOHA contaminants

	OR	95% CI	*p*-value
Biological dust	1.07	0.96 – 1.18	0.21
**Mineral dust**	**1.12**	**1.01 – 1.24**	**0.04**
Gas fumes	1.1	0.99 – 1.24	0.08
**Vapors, Dusts, Gases, Fumes**	**1.15**	**1.02 – 1.30**	**0.03**
All pesticides	1.11	0.93 – 1.33	0.24
Fungicides	1.04	0.87 – 1.25	0.65
Insecticides	1.15	0.94 – 1.41	0.18
Aromatic solvents	1.07	0.97 – 1.19	0.17
Chlorinated solvents	1.01	0.9 – 1.13	0.89
Other solvents	1.05	0.95 – 1.17	0.32
Metals	1.07	0.96 – 1.19	0.25

B. DOM contaminants

	OR	95% CI	*p*-value
**Asbestos**	**1.12**	**1.01 – 1.23**	**0.03**
Chromium	1.04	0.91 – 1.19	0.58
Diesel motor exhaust	1.08	0.96 – 1.22	0.21
Nickel	1.01	0.85 – 1.19	0.94
Polycyclic aromatic hydrocarbons	1.19	0.98 – 1.44	0.08
Silica	1.06	0.95 – 1.17	0.3
Animals	1.03	0.90 – 1.18	0.71
Biological dust	1.04	0.98 – 1.11	0.23
Endotoxin	1.05	0.93 – 1.17	0.43

Table S8

Organ manifestations per patient with IgG4-RD. IgG4-associated cholangitis (IAC), Autoimmune pancreatitis (AIP), IgG4-related sialadenitis (SIAL), Retroperitoneal fibrosis (RPF), IgG4-related kidney disease (KID), IgG4-related prostatitis (PROS), IgG4-related thyroiditis (THYR), IgG4-related ophthalmic disease (LACR), IgG4-related aortitis (AOR), IgG4-related hypophysitis (HYPO), IgG4-related lung disease (LUNG).

ID	Manifestations	ID	Manifestations	ID	Manifestations	ID	Manifestations
12126	IAC, AIP	45764	AIP	45764	AIP	80256	IAC, AIP
12642	AIP	46600	IAC	46600	IAC	80613	IAC, AIP
12690	IAC, AIP	47824	IAC	47824	IAC	82339	IAC, AIP
13756	AIP	49253	AIP	49253	AIP	83216	IAC
15514	IAC	49619	AIP	49619	AIP	83504	AIP
17139	AIP	50522	IAC	50522	IAC	84251	AIP, SIAL
17557	IAC, AIP	50914	IAC, AIP	50914	IAC, AIP	86901	IAC, SIAL, THYR
18008	IAC, AIP	51573	AIP	51573	AIP	88372	IAC
18136	IAC	51695	AIP	51695	AIP	88613	IAC
18201	IAC	51740	IAC	51740	IAC	89339	AIP
18513	IAC	51918	IAC, AIP	51918	IAC, AIP	89858	IAC
21113	IAC, AIP	52268	IAC	52268	IAC	90158	AIP
21646	AIP	52273	IAC	52273	IAC	91179	IAC, AIP, PROS
21707	IAC, AIP, SIAL	53869	AIP	53869	AIP	91389	AIP
23037	IAC, AIP	53888	IAC, AIP	53888	IAC, AIP	93527	IAC, AIP, SIAL, KID
23429	IAC	53940	AIP	53940	AIP	94052	IAC, RPF
26300	IAC	54983	IAC, AIP, AOR	54983	IAC, AIP, AOR	94485	IAC, AIP
26825	AIP	57580	IAC, AIP, RPF	57580	IAC, AIP, RPF	95051	IAC
26927	AIP	57973	IAC, AIP, SIAL, LACR	57973	IAC, AIP, SIAL, LACR	95285	IAC, AIP
27302	IAC	58087	AIP	58087	AIP	95886	AIP
27423	IAC, AIP, SIAL	59386	IAC, AIP	59386	IAC, AIP	96143	IAC, AIP, SIAL
27492	IAC, AIP, LUNG, KID	59695	IAC, AIP, RPF, PROS	59695	IAC, AIP, RPF, PROS		
29071	IAC, AIP, SIAL, LACR	59871	IAC	59871	IAC		
30723	AIP	61178	IAC	61178	IAC		
31914	IAC	62351	IAC, AIP	62351	IAC, AIP		
32840	AIP	63126	IAC, KID	63126	IAC, KID		
32871	IAC, AIP	63740	IAC, AIP	63740	IAC, AIP		
34176	IAC, AIP, KID	64334	IAC, AIP	64334	IAC, AIP		
34188	IAC	65745	IAC, AIP	65745	IAC, AIP		
34587	AIP	67198	IAC	67198	IAC		
35378	IAC, AIP, PROS	68709	IAC, AIP	68709	IAC, AIP		
35386	IAC, AIP	69044	AIP	69044	AIP		
37806	AIP	69192	IAC, AIP	69192	IAC, AIP		
39261	AIP	71345	AIP	71345	AIP		
40774	IAC, AIP	72501	AIP	72501	AIP		
42026	IAC, AIP	73120	IAC, AIP	73120	IAC, AIP		
43317	AIP, RPF, AOR	73228	IAC, AIP	73228	IAC, AIP		
43800	IAC, AIP	75285	IAC, AIP	75285	IAC, AIP		
45351	IAC, AIP	77052	IAC, AIP	77052	IAC, AIP		
45554	AIP, SIAL, HYP	78126	AIP	78126	AIP		
